# Health care resource utilisation in primary care prior to and after a diagnosis of Alzheimer’s disease: a retrospective, matched case–control study in the United Kingdom

**DOI:** 10.1186/1471-2318-14-76

**Published:** 2014-06-17

**Authors:** Lei Chen, Catherine Reed, Michael Happich, Allen Nyhuis, Alan Lenox-Smith

**Affiliations:** 1 Eli Lilly and Company, Lilly Corporate Center, Indianapolis IN 46285, USA; 2 Lilly UK, Erl Wood Manor, Windlesham, Surrey GU20 6PH, UK; 3 Lilly UK, Lilly House, Priestley Road, Basingstoke, Hampshire RG24 9NL, UK

**Keywords:** Alzheimer’s disease, Dementia, United Kingdom, Primary care

## Abstract

**Background:**

This study examined medical resource utilisation patterns in the United Kingdom (UK) prior to and following Alzheimer’s disease (AD) diagnosis.

**Methods:**

A patient cohort aged 65 years and older with newly diagnosed AD between January 2008 and December 2010 was identified through the UK’s Clinical Practice Research Datalink (CPRD). Patients with a continuous record in the CPRD (formerly the General Practice Research Database [GPRD]) for both the 3 years prior to, and the 1 year following, AD diagnosis were eligible for inclusion. A control cohort was identified by matching general older adult (GOA) patients to patients with AD based on year of birth, gender, region, and Charlson Comorbidity Index at a ratio of 2:1. Medical resource utilisation was calculated in 6-month intervals over the 4-year study period. Comparisons between AD and GOA control cohorts were conducted using conditional logistic regression for patient characteristics and a generalised linear model for resource utilisation.

**Results:**

Data for the AD cohort (N = 3,896) and matched GOA control cohort (N = 7,792) were extracted from the CPRD. The groups were 65% female and the AD cohort had a mean age of 79.9 years (standard deviation 6.5 years) at the date of diagnosis. Over the entire study period, the AD cohort had a significantly higher mean primary care consultation rate than the GOA cohort (p < .0001). While the GOA cohort primary care consultation rate gradually increased over the 4-year period (ranging from 5 to 7 consultations per 6-month period), increases were more pronounced in the AD cohort (ranging from 6 to 11 consultations per 6-month period, peaking during the 6-month periods immediately prior to and post diagnosis). The AD cohort also had a higher overall specialty referral rate than the GOA cohort over the 4-year period (37% vs. 25%, respectively; p < .0001); the largest difference was during the 6 months immediately prior to AD diagnosis (17% vs. 5%, respectively; p < .0001).

**Conclusions:**

In the UK, AD diagnosis is associated with significant increases in primary and secondary care resource utilisation, continuing beyond diagnosis. This evidence may be important to health care commissioners to facilitate effective mobilisation of appropriate AD-related health care resources.

## Background

Across Europe, the 6.3 million patients living with dementia impose a high financial burden. With a total annual health care cost of €16.95 billion (€2673 per patient) and a total non-medical cost of €88.2 billion (€13,911 per patient), the impact of dementia on the financial health of Europe should not be under-estimated [1]. Alzheimer’s disease (AD) is the most common form of dementia, and accounts for over 60% of all cases [2]. In the United Kingdom (UK) about 400,000 patients have AD [2], and the incidence rates double as age increases by 5 years (after 75 years of age) [3]. While 1 person in 88 of the entire UK population has a form of dementia, that ratio jumps to 1 person in 14 over age 65, and to 1 person in 6 over age 80 [2].

Clinical diagnosis of probable AD, like many neurocognitive disorders, relies on examination of the patient’s physical and mental state in consultation with a close friend or relative of the patient and requires identification of a cognitive abnormality alongside inability of the patient to carry out daily living activities [4,5]. The subjective process of diagnosing dementia or AD often results in a lengthy gap between symptom onset and diagnosis [6]. In a recent survey of UK patients with dementia, 68% had experienced a 1-year or longer delay in diagnosis, while 8% reported a delay of 5 years or longer [7]. Although recent advances in brain imaging along with the identification of distinctive and reliable biomarkers [4] indicate potential for the diagnostic process to be more objective, there are issues of resources and reliability to be considered before these become part of routine care.

There are currently no treatments available that can alter the progressive course of dementia. However, there are treatments that can assist in symptom management [2]. For patients with mild to moderate AD, three acetylcholinesterase (AChE) inhibitors are available (donepezil, galantamine, and rivastigmine). Memantine is available for patients with moderate to severe AD [8] and antidepressants, antipsychotics, and anxiolytics have been used to treat psychological symptoms [2].

The cost of dementia care varies greatly depending on the stage of the disease; in some studies the cost of care was more than doubled between mild and severe dementia [9,10]. Patients with dementia also have higher rates of hospitalisation with longer duration of stay [11]. In fact, at any given time, one-quarter of all hospital beds in the UK are occupied by patients with dementia [12].

The burden of dementia and AD on the primary care sector is not well understood [13]. Studies in the United States (US) [13], the Netherlands [14], and Germany [15] found that the number of primary care/ambulatory care consultations (or the cost of that care) increased prior to diagnosis and remained high immediately following diagnosis. Other studies in Denmark [11], the US [16], and France [17] found varying or even no differences in primary care resource utilisation in comparisons among patients with AD with differing degrees of impairment, and versus non-AD dementia or control cohorts without dementia. Strikingly, there are currently no published studies that investigate the pattern of resource utilisation before AD diagnosis in the UK.

In this retrospective cohort study, the goal was to characterize the longitudinal pattern of medical resource utilisation of individuals with AD prior to, and after, their diagnosis and to compare those patterns with the resource utilisation of matched control individuals, a general older adult patient population without AD.

## Methods

### Data source

This study used electronic medical record data from the UK Clinical Practice Research Datalink (CPRD), which has evolved from the General Practitioner Research Database (GPRD). The GPRD was established in 1987, and data are gathered in a non-interventional way from the records of general practitioners in the UK. The anonymised patient information includes demographics, diagnoses, comorbidities, and prescribing information. A detailed description of CPRD is available elsewhere [18-20].

CPRD has an established linkage between GPRD and the Hospital Episode Statistics (HES) which was also used in this study. The HES [21] is a data warehouse containing details of all admissions to National Health Services (NHS) hospitals in England including acute hospitals, primary care trusts, and mental health trusts. The HES contains admitted patient care data from 1989 onwards, and processes more than 125 million records each year.

### Sample selection

This study used an incidence-based, matched case–control design. Two cohorts of patients 65 years and older were identified: (1) those who received a first diagnosis of AD (AD cohort), and (2) a general older adult cohort of patients who did not have a diagnosis of AD or dementia (GOA cohort).

We identified patients who had the first diagnosis of AD between 01 January 2008 and 31 December 2010. We defined the index date as the first diagnosis of AD for a given patient. Patients 65 years or older old at the index date were included in the AD cohort. Patients also needed to have a case history for at least 3 years before, and 1 year after, the index date. We excluded those who had ever received a diagnosis of early-onset AD, or had a non-AD dementia diagnosis (including vascular dementia, dementia with Lewy bodies, and frontotemporal dementia) during the post-index period.

For each patient in the AD cohort, 2 general older adults without an AD or other dementia diagnosis were matched on the basis of year of birth, gender, region, and comorbidity severity as measured by Charlson Comorbidity Index (CCI) [22]. Since the studied condition is AD, a type of dementia, our calculation of CCI omitted dementia and only included a total of 16 physical conditions that represent common conditions like cancer, diabetes mellitus, and cardiovascular disease. The matched GOA individuals were also required to have a complete case history for the corresponding 4-year study period. If more than 2 patients met the matching criteria, 2 matched individuals were selected randomly.

Ethics approval for this study was given by the Independent Scientific Advisory Committee (ISAC) at CPRD in 2012 (protocol number: 12-071R).

### Patient characteristics

The AD and GOA cohorts were evaluated for the following characteristics: (1) demographics (age, sex, region); (2) baseline rates of selected comorbidities (hypertension, depression, psychosis); and (3) baseline CCI score.

### Measures of medical resource utilisation

The overall consultation rate was calculated as the sum of all recorded consultations excluding administrative consultations. More than one consulting record per day was counted as one consultation [23]. Consultations were classified into the following mutually exclusive categories: ‘GP (general practice) consultation’ (including consultations coded as clinic, surgery, and emergency consultation), ‘house call’ , ‘telephone consultation’ , ‘third-party consultation’ (friend, family, or guardian contact on behalf of patient), and ‘other’.

The overall specialist referral rate was calculated, as were as referrals to psychiatrists/memory clinics, neurologists, and geriatrics. In the UK, AD is often diagnosed by specialists in a memory clinic [2]. As these specialists usually have a psychiatric background — 79% of UK investigators in the GERAS study were psychiatrists who specialize in the care of elderly patients [24] — clinic visits are typically coded as a psychiatric referral. AD-related drug utilisation rates (including AChE inhibitors, memantine, antidepressants, antipsychotics, and anxiolytics/sedatives) were measured. Hospital admissions were estimated based on the HES-eligible subpopulation. The length of hospitalisation was calculated by subtracting the admission date from the discharge date for days within the study period. This excluded day case, which has a length of stay of zero days.

### Data/statistical analyses

Descriptive statistics were presented by study cohort and compared using a conditional logistic regression for non-matched variables. To understand the longitudinal pattern of the resource utilisation, the 4-year study period was divided into eight 6-month intervals: six pre-index intervals (for a total of 3 years prior) and 2 post-index intervals (for a total of 1 year post). Resource utilisation was calculated for each interval. The resource utilisation between the AD and GOA cohorts was first compared using t-tests, chi-square tests, and Wilcoxon rank-sum tests depending on the data type and distribution. To account for the matched-case control study design, as well as to adjust for the difference in individual comorbidities, resource utilisation data were assessed using a generalised linear model that accounted for correlation among repeated measures. The models adjusted for differences in patient characteristics, such as comorbidities of hypertension and depression. Psychosis was not included as a covariate in the model because the prevalence of this baseline diagnosis was very low in both study cohorts.

All analyses were conducted using commercially available statistical software (SAS version 9.2; SAS Institute Inc., Cary, North Carolina). P-values < .05 were considered statistically significant.

## Results

### Cohort characteristics

**Figure 1:**
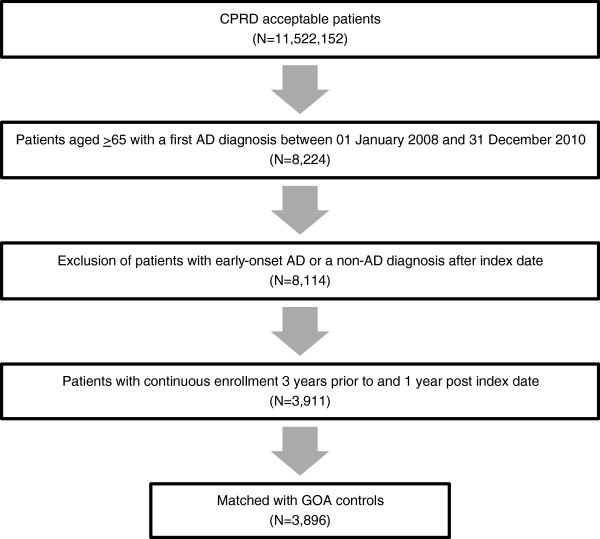
**Patient data identification from the CPRD.** Clinical Practice Research Datalink (CPRD) records identified patients with Alzheimer’s disease (AD) diagnosed between 01 January 2008 and 31 December 2010, and a matched general older adult (GOA) cohort. Excluding ineligible patients produced a cohort of 8,114 patients; 3,911 had continuous CPRD records throughout the 4-year study period and 3,896 were matched 1:2 with 7,792 GOA controls by birth year, gender, region, and comorbidity.

The AD cohort included 3,896 patients who were matched 1:2 with 7,792 GOA control patients (Figure 1). Of these, 1,785 patients (45.8%) in the AD cohort and 3,407 patients (43.7%) in the GOA cohort comprised the HES subpopulation. As a result of the case-control design, the mean age (79.9 years), gender (65% female), CCI (mean 0.69 and 36.2% with CCI ≥ 1), and geography (73% from England) were the same for both groups (Table 1). More patients in the AD cohort than in the GOA cohort had reported depression (16.5% vs. 9.2%; p < .0001) and psychosis (0.31% vs. 0.04%; p = .0013); in contrast, fewer patients in the AD cohort had hypertension compared with GOA patients (9.3% vs. 10.5%; p = .0351) (Table 1). In the HES subpopulation, AD and GOA cohorts were comparable in terms of age, gender, and geographic region. However, HES-eligible AD cohort patients were significantly less likely to have at least one chronic physical condition (i.e., CCI ≥ 1) than GOA patients (37.4% vs. 39.4%; p = .0332).

**Table 1:** Cohort characteristics

	**Patients with AD N = 3,896**	**GOA Controls N = 7,792**	**P-value**
Age, mean years (standard deviation)	79.9 (6.5)	79.9 (6.5)	
Age Group, % of patients			
65-69	7.3%	7.3%	
70-74	13.7%	13.7%	
75-79	25.7%	25.7%	
80-84	27.7%	27.7%	
85+	25.7%	25.7%	
Female,% of patients	65.2%	65.2%	
Geographic Region, % of patients			
England	73.2%	73.2%	
Northern Ireland	3.5%	3.5%	
Scotland	14.6%	14.6%	
Wales	8.7%	8.7%	
CCI^a^, mean (range)	0.69 (0, 6)	0.69 (0, 6)	
CCI ≥ 1, % of patients	36.2%	36.2%	
Hypertension, % of patients	9.3%	10.5%	.0351
Depression, % of patients	16.5%	9.2%	<.0001
Psychosis, % of patients	0.31%	0.04%	.0013

AD = Alzheimer’s disease; GOA = general older adult; CCI = Charlson Comorbidity Index.

^a^CCI was calculated excluding dementia component.

### Consultation rates

**Figure 2:**
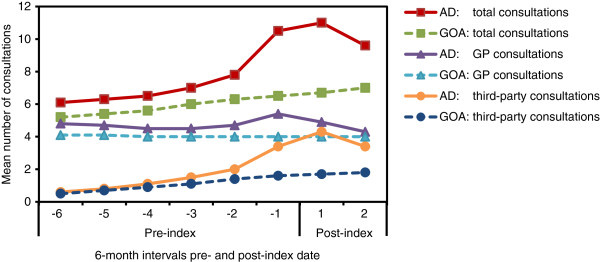
**Mean numbers of consultations per person.** For total consultations, AD vs. GOA comparisons were significant (p < .0001) at every time interval. The x-axis values represent 6-month intervals pre- and post-index (AD diagnosis) date. AD = Alzheimer’s disease; GOA = general older adult; GP = general practise.

Over the 4-year study period, the mean overall number of total primary care consultations was significantly higher in the AD cohort compared with GOA patients (64.8 vs. 48.7 overall total consultations; p < .0001). Total consultations in the GOA cohort gradually increased over this period. In the initial 6-month period, the mean number of total consultations in the AD cohort was significantly higher than in the GOA cohort (6.1 vs. 5.2 total consultations; p < .0001) (Figure 2). The AD cohort then showed a sharp increase (to 10.5 total consultations) during the 6-month period prior to diagnosis; the rate remained high post diagnosis, and was significantly higher than the GOA cohort (p < .0001) (Figure 2).GOA patients had a stable GP consultation rate over the 4-year study period (approximately 4 consultations per 6 months), whereas the AD cohort had a slightly higher GP consultation rate that was relatively stable throughout the 4-year period except for a peak (to 5.4 GP consultations) occurring in the 6 months immediately prior to diagnosis (Figure 2).Similar trends were observed for third-party consultations. The overall mean number of third-party consultations was significantly higher in the AD cohort than for GOA patients (17.0 vs. 9.7 overall third-party consultations; p < .0001). In the initial 6-month period, the mean number of third-party consultations for the AD cohort was significantly higher compared with the GOA cohort (0.6 vs. 0.5 third-party consultations; p = .0016) (Figure 2). Over the 4-year study period, third-party consultations increased steadily for GOA patients, whereas the AD cohort showed an increase (to 3.4 third-party consultations) in the 6-month period prior to diagnosis that further increased for a peak (4.3 third-party consultations) in the first post-diagnosis period (Figure 2).

### Specialty referral rates

**Figure 3:**
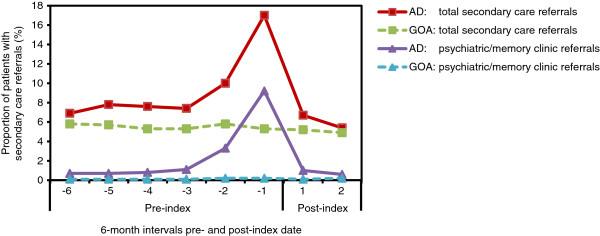
**Proportion of patients with at least one new specialty referral.** For total secondary care specialty referrals, AD vs. GOA comparisons were significant (p < .02) at every time interval except the second post-index interval. The x-axis values represent 6-month intervals pre- and post-index (AD diagnosis) date. AD = Alzheimer’s disease; GOA = general older adult.

The proportion of patients with at least one secondary care specialty referral over the 4-year period was also significantly higher in the AD cohort compared with GOA patients (36.8% vs. 24.8%; p < .0001). While the overall specialty referral rates were significantly higher in the AD cohort compared with GOA patients for neurology (1.0% vs. 0.5%; p = .0022) and geriatrics (3.1% vs. 1.1%; p < .0001) referrals, the majority of the increase in the total specialty referral rate for patients with AD can be attributed to the high number of psychiatry referrals (Figure 3). During the first 6-month period, 0.67% of patients in the AD cohort received a psychiatry specialty referral, which increased to a peak of 9.2% in the 6 months immediately preceding diagnosis. In comparison, psychiatry referrals remained stable in the GOA cohort throughout the study at about 0.1% per 6-month period (p < .0001 vs. AD cohort in all 6-month periods) (Figure 3).

### Prescription rates

**Figure 4:**
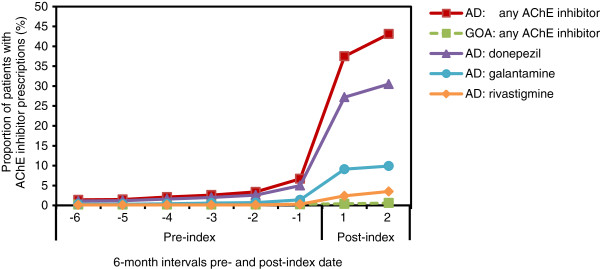
**Proportion of patients with at least one prescription for an AChE inhibitor.** For total AChE inhibitor prescriptions, AD vs. GOA comparisons were significant (p < .0001) at every time interval. The x-axis values represent 6-month intervals pre- and post-index (AD diagnosis) date. AChE = acetylcholinesterase; AD = Alzheimer’s disease; GOA = general older adult.

In the first post-diagnosis 6-month period, 37.5% of patients in the AD cohort were prescribed at least one AChE inhibitor (Figure 4). Unsurprisingly, due to the way the cohort was defined, only 0.67% of the GOA cohort had been prescribed an AChE inhibitor over the 4-year study period. In contrast, 46.6% of patients in the AD cohort had been prescribed at least one AChE inhibitor (p < .0001). The AD cohort was also significantly more likely than the GOA cohort to have been prescribed memantine (2.5% vs. 0.13%; p = .0024), antidepressants (41.4% vs. 23.2%; p < .0001), and antipsychotics (23.7% vs. 14.5%; p < .0001). However, there was no significant difference between AD and GOA cohorts in the proportion of patients prescribed anxiolytics (26.6% vs. 19.8%; p = .2178).

### Hospitalisation rates

**Figure 5:**
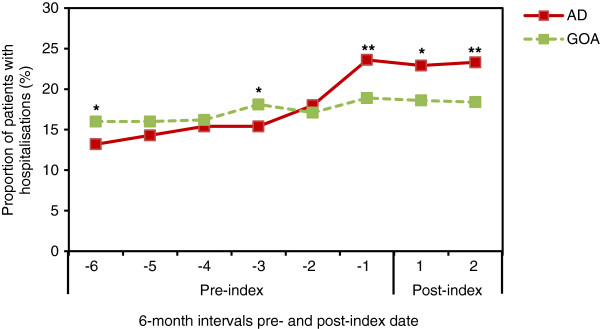
**Proportion of patients with at least one hospital admission.** HES-eligible patients included n = 1,785 for the AD cohort and n = 3,407 for the GOA cohort. For AD vs. GOA comparisons, *p ≤ .01 and **p < .0001. The x-axis values represent 6-month intervals pre- and post-index (AD diagnosis) date. AD = Alzheimer’s disease; GOA = general older adult; HES = Hospital Episode Statistics.

In the HES subpopulation, patients in the AD cohort were significantly less likely to be hospitalised than were HES-eligible GOA patients during the first 6-month study period (13.2% vs. 16.0%; p = .0062) (Figure 5). However, patients in the AD cohort were significantly more likely to be hospitalised than GOA patients in the 6 months immediately prior to diagnosis (23.6% vs. 18.9%; p < .0001). The higher rate of hospitalisation for the AD cohort versus the GOA cohort continued through the final 6-month study period (23.3% vs. 18.4%; p < .0001) (Figure 5). The duration of hospitalisation was also increased; in the 6 months preceding AD diagnosis the mean length of hospital stay for patients with AD was more than twice that of GOA patients (1.9 days vs. 0.79 days; p < .0001). The length of hospitalisation remained significantly higher in the AD cohort versus the GOA cohort for the year following AD diagnosis (p < .0001).

## Discussion

This population- and incidence-based, matched case-control study found that the burden on primary care resources is higher in patients with AD versus their demographic- and comborbidity-comparable elderly control counterparts. The higher rates of primary care resource utilisation are evident in the AD cohort relative to GOA control patients as much as 3 years prior to AD diagnosis, with a sharp increase in the AD cohort immediately preceding the AD diagnosis that is maintained in the one year following diagnosis. This is the first study to report such a finding in the UK, and is consistent with results of other studies [13-15]. In Albert *et al.*, the burden was measured by health care expenditures [13], while Ramakers *et al.* and Eisele *et al.* both used primary care consultation rates as a measure of health care burden [14,15]. There are, however, some striking differences between the various study populations. Two of the studies [13,14] included fewer than 100 patients with prodromal AD, while one study [15] had an AD cohort that was approximately half the size of that used for the current study.

The initial primary care consultation rate observed for the GOA control cohort in the current study (approximately 11 consultations per year) was consistent with general medical utilisation patterns as documented in a recent UK Department of Health report [25]. Although this study does not reveal why patients were seeking primary care consultations, it is reasonable to assume that the increases in consultation rates and referrals for patients in the AD cohort reflect the diagnostic process. If so, diagnosis will be accompanied by an increase in health care utilisation regardless of when it occurs. Another possibility is that patients with undiagnosed AD may be less able to maintain healthy habits, such as regular medication regimens. Thus, the peak in primary care utilisation may reflect general health deterioration as the patients’ daily living skills are increasingly compromised. It is also possible that these visits may be due to sequelae subsequent to the AD, such as gait imbalances [13]. It is notable that third-party consultations (by guardians/family/friends on behalf of the patient) as well as hospitalisations both increased, suggesting that both the diagnostic pathway and general medical decline may actively contribute to the observed increase in primary care utilisation. Further analysis is needed to elucidate the specific contribution of the AD diagnostic pathway versus medical needs.

This study also found that the AD cohort was more likely to have a diagnosis or reported symptoms of depression and/or psychosis, although the prevalence of psychosis was lower than expected in both cohorts. Recent estimates are of about 1% or higher of older-age adults exhibiting psychotic symptoms [26,27]. The low rates of psychosis in our study and the contrasting higher antipsychotic prescriptions rates in the AD cohort versus GOA patients may be a result of prescriptions originating in a secondary care setting or prescribing to patients in the absence of a psychosis diagnosis. Our study also found lower rates of hypertension in the AD cohort than the control cohort, which is surprising since hypertension is a known risk factor for AD. In contrast, a German study reported higher rates in the AD cohort than controls [15]. Notably, though, the proportions of patients with hypertension in the GOA and AD cohorts in our study were numerically close — statistical significance of the small difference between cohorts could be an artifact of the sample size — and may warrant further study to understand the reason(s) for the observed rates.

The initial secondary care specialty referral rates for the AD and GOA cohorts (6.9% and 5.8% in the first 6-month period, respectively; if extrapolated these would each be about 12% to 14% per year) were comparable with previously published referral rates for the GPRD (13.9% per year) [28]. The referral rates for the AD cohort were higher than the GOA cohort throughout the study, and sharply increased in the AD cohort in the 6-month period immediately preceding diagnosis (to 17.0%); this was largely driven by an increase in psychiatric/memory clinic referrals in this time period before diagnosis.

While GP practitioners are the cornerstone of medical care, they have varying familiarity with AD and high demands on their time and attention that may limit their capacity to manage AD care. For this reason, the NHS has established memory clinics throughout the UK and recommends that AD be diagnosed within these clinics or by a psychiatric specialist, since any prescribed pharmacological therapy (e.g., AChE inhibitor) should be initiated by a specialist. Per treatment guidelines, such clinics should be the single point of referral for possible dementia diagnosis [8]. However, this study found that about 90% of patients with an AD diagnosis in their medical record were not referred to a psychiatry/memory clinic specialist by their GP provider. It is likely that some patients were referred by non-GP physicians, such as during an unrelated hospital admission, although some of these AD diagnoses were perhaps initiated directly by the GP practitioner. As a result, there may be variability around the exact criteria used for initial diagnosis and extent of disease progression at that time.

There is growing interest in the quick, efficient diagnosis and early intervention for AD, considering the high resource utilisation prior to and post diagnosis (in the current diagnostic pathway) may be related to both the progressive nature of the disease and the often lengthy diagnosis process. Albert *et al.* calculated that undiagnosed AD is responsible for $130 million to nearly $200 million in excess cost to the US each year [13]. While the number of patients with AD is much smaller in the UK than in the US, the increase in medical consultations is likely to impose a significant financial burden on the UK. There is keen interest in early treatment of neurocognitive diseases, as treatments may be most effective when structural changes to the brain are minimal [5]; this is also reflected in the large number of compounds under development for the treatment of early-stage AD. In addition, emergence of new clinical tools may provide the means to increase the efficiency of diagnosis, including reducing rates of false-positive and false-negative diagnoses that can contribute to the high cost and resource consumption of primary care of dementia patients [29].

Reassuringly, nearly 50% of the AD cohort was prescribed an AChE inhibitor within 1 year of diagnosis, adhering to the 2012 NICE treatment guidelines recommending treatment for patients with both mild and moderate to severe AD [8]. While it is unclear at this time whether earlier diagnosis of AD would subsequently decrease the burden on the health care system, there is evidence that early AD diagnosis decreases subsequent primary health care medical costs [30]. Our study did not address the timing of AD diagnosis as a factor in the health care resource burden on primary care. However, given the current challenges in the diagnostic pathway, combined with our evidence of the significant burden this places on primary care, it is reasonable to hypothesise that increasing the efficiency of diagnosis could improve resource and cost management for GP providers. This might be accomplished in several ways, including shortening the duration and/or streamlining the process from the time of onset of AD symptoms in the GP setting to subsequent diagnostic confirmation and mobilisation of appropriate care.

This study relied entirely on electronic medical records; eligible patients were identified from over 11 million patients in the CPRD. This population-based approach should provide a cross-section of rural/urban location, race, and socio-economic status, avoiding many biases inherent in studies focused on a specific region or clinic. There were 11,688 patients in the study population, a far larger sample size than is easily obtainable from in-person interviews. Most importantly, the resource utilisation patterns described herein occurred in the real-world context of diverse UK communities. The longitudinal design was an advantage, as it followed patients for the 3-year period prior to AD diagnosis and 1-year post. This is important in the context of typical delays that occur between the onset of AD symptoms and obtaining an AD diagnosis [6,7].

The results of this study should be interpreted in the context of its limitations. First, while electronic medical records data are extremely valuable for efficient and effective examination of health care resource utilisation, there exist inherent limitations. Data are collected from routine practice; thus, some data may be missing and coding errors might have occurred. Second, the study analysed the CPRD database of electronic medical records for GP providers. Resource utilisation that occurred at the specialist level may not have been captured in the system, as the database does not link patient records to all specialist secondary care information (except HES). The lack of complete secondary care information limited our ability to draw conclusions about the role of the AD diagnostic pathway (versus disease progression) in resource utilisation. Future studies should include evaluation of resource utilisation at the specialist level. Third, we were also limited by the study design; inclusion in the study required a documented AD diagnosis, so patients who did not visit a physician or did not receive a diagnosis were not included in the study population. Lastly, this study matched the AD cohort with a general elderly control population by demographics and comorbidity index and adjusted for potential available confounders, but an unmeasured confounder may exist.

This study clearly shows that in the UK, AD imposes a burden on the primary care system, particularly around the time of diagnosis. Given the important role of GPs in the lives of their patients, this finding is not surprising, and is unlikely to change in the future. Because of their long-term relationships with patients, GPs are uniquely placed to observe cognitive changes in their patients over time. However, it is important to recognise the additional stress that this places on the primary care system.

## Conclusions

Our study is the first to analyse the longitudinal pattern of medical resource utilisation among patients with AD in the UK. It shows a clear increase in primary care consultations, especially in the 6 months prior to AD diagnosis and in the year post diagnosis, suggesting AD imposes a substantial resource burden on UK primary care. Additional research is necessary to estimate costs specifically attributable to the diagnostic pathway. Economic evaluations that address components of diagnostic procedures, including those taking place at the specialist level, would be valuable additions to the existing body of research on resource utilisation patterns in patients with AD. This would provide evidence to health plan sponsors who want to facilitate mobilisation of appropriate AD-related health care resources in a more time- and cost-effective manner. Ultimately, this would ensure the efficient provision of resources for AD diagnosis and treatment and potentially alleviate the burden on primary care.

## Abbreviations

AChE: Acetylcholinesterase; AD: Alzheimer’s disease; CCI: Charlson Comorbidity Index; CPRD: Clinical practice research datalink; GP: General practise; GOA: General older adult; GPRD: General practice research database; HES: Hospital episode statistics; ISAC: Independent Scientific Advisory Committee; NHS: National Health Services; SD: Standard deviation; UK: United Kingdom; US: United States.

## Competing interests

All authors are employees and minor shareholders of Eli Lilly and Company and/or one of its subsidiaries.

## Authors’ contributions

LC conceived the study concept and acquired data. CR, MH, and ALS designed and interpreted the study. AN performed statistical analyses. All authors were involved in critical revision of this manuscript and provided final approval prior to submission.

## Pre-publication history

The pre-publication history for this paper can be accessed here:

http://www.biomedcentral.com/1471-2318/14/76/prepub
